# SFPQ, a multifunctional nuclear protein, regulates the transcription of *PDE3A*

**DOI:** 10.1042/BSR20170975

**Published:** 2017-08-09

**Authors:** Dong Keun Rhee, Steven C. Hockman, Sunkyung Choi, Yong-Eun Kim, Chungoo Park, Vincent C. Manganiello, Kee K. Kim

**Affiliations:** 1 Laboratory of Biochemical Physiology, National Heart, Lung, and Blood Institute, National Institutes of Health, Bethesda, MD 20892, U.S.A.; 2 Department of Biochemistry, Chungnam National University, Daejeon 34134, Republic of Korea; 3 School of Biological Sciences and Technology, Chonnam National University, Gwangju 61186, Republic of Korea

**Keywords:** Cervical cancer, DNMDP, PDE3A, SFPQ, Transcription

## Abstract

Phosphodiesterase 3A (PDE3A), a member of the cGMP-inhibited cyclic nucleotide phosphodiesterase (PDE) family, plays important roles in oocyte maturation and vascular smooth muscle cell proliferation. However, the molecular mechanisms that regulate *PDE3A* gene expression remain largely unknown. In the present study, we investigated the transcriptional regulation of *PDE3A*, and found that the splicing factor proline- and glutamine-rich (SFPQ) protein modulated *PDE3A* mRNA levels. Multiple transcription start sites (TSS1, 2, and 3) were identified within the first exon of *PDE3A* using 5′-rapid amplification of cDNA ends (RACE). Variable expression levels of three *PDE3A* variants were also observed in human tissues and HeLa cells. Several putative SFPQ-binding sites were identified upstream of the regulatory region of *PDE3A*-TSSs using ChIP sequencing (ChIP-seq). Serum-induced *PDE3A* expression was affected by increasing the amount of SFPQ binding to the upstream regulatory region of *PDE3A*. In addition, transcription of *PDE3A* was lower in human cervical adenocarcinoma cells compared with normal cervical tissue. Furthermore, overexpression of *PDE3A* induced sensitivity to anticancer therapeutic agent, 6-(4-(diethylamino)-3-nitrophenyl)-5-methyl-4,5-dihydropyridazin-3(2H)-one (DNMDP), in HeLa cells. Taken together, these results suggest that SFPQ functions as a transcriptional activator of *PDE3A*, which is involved in the regulation of DNMDP sensitivity, offering a novel molecular target for the development of anticancer therapies.

## Introduction

To maintain the normal cellular physiology, tight regulation of cAMP and cGMP signaling is pivotal, causing serious diseases if disrupted [1,2]. By catalyzing the hydrolysis of cAMP and cGMP, cyclic nucleotide phosphodiesterases (PDEs) regulate their own intracellular concentrations and properly modulate important downstream signaling pathways [1,2]. The PDE superfamily consists of 11 gene families of isoenzymes (PDEs 1–11). Over 100 PDE isoforms are reportedly expressed based upon the analysis of multiple ORFs, promoters, and alternative splicing patterns. PDEs regulate discrete aspects of intracellular signaling through spatial and functional diversification, and their dysfunction affects numerous clinically relevant pathways linked to human diseases [1,3,4]. The localization of individual PDEs to specific intracellular sites or molecular complexes helps regulate the compartmentation of cyclic nucleotide signaling [3,5].

The PDE3 family contains two subfamilies, which are encoded by highly related genes on chromosomes 12 (*PDE3A*) and 11 (*PDE3B*) [4]. Amongst the 11 PDE families, PDE3 contains unique transmembrane domains [1,6]. PDE3A is expressed in the cardiac tissue and vascular smooth muscle, platelets, oocytes, kidneys, and cervix. It is also expressed in numerous cancer cell lines, including HeLa cells and A549 cells. PDE3A has been reported to play critical roles in myocardial contractility, platelet aggregation, vascular smooth muscle contraction, vascular myocyte proliferation, oocyte maturation, and regulation of renin release [1,7–9]. Maass et al. [10] reported that missense mutations in *PDE3A* increase PKA-induced PDE3A phosphorylation and cAMP hydrolytic activity, causing hypertension and brachydactyly type E. In addition, several studies suggest that the intergenic regions of *hPDE3A* may be useful drug targets to improve anti-TNF therapy in rheumatoid arthritis, HDL cholesterol levels, and type 2 diabetes mellitus [11,12]. Recently, Waal et al. reported that human PDE3A is a modulator for 6-(4-(diethylamino)-3-nitrophenyl)-5-methyl-4,5-dihydropyridazin-3(2H)-one (DNMDP)-induced cell death [8^8^. Furthermore, DNMDP cytotoxicity is regulated by hPDE3A expression levels and not by hPDE3A-mediated cAMP hydrolysis [8].

The gene expression cascade from chromatin to mRNA translation to protein decay is a dynamic and complex process in mammalian cells [13,14]. Most mammalian genomes carry fewer genes than other eukaryotes, which indicate that complex mechanisms are likely to exist in mammals to increase the number of functional isoforms originating from a single gene [15]. In mammalian cells, pre-mRNA alternative splicing mechanisms, such as alternative promoter selection, are common ways to generate diversity of gene products [15,16]. Pre-mRNA alternative splicing changes the structure, intracellular localization, and binding partner interactions of proteins [16,17]. A previous study of human myocardial PDE3A [18] demonstrated that two isoforms of PDE3A (hPDE3A) are generated. Specifically, *hPDE3A-isoform 1* (NM_000921.4) is the longer transcript, while *hPDE3A-isoform 2* (NM_001244683.1) has a truncated 5′-UTR and 5′-coding region. Three hPDE3A protein variants (hPDE3A1/2/3) generated by *hPDE3A-isoform 1* have been suggested to have different subcellular localizations in human myocardial cells and distinct roles in selective phosphorylation of protein targets [19]. Recently, it has been reported that splicing and degradation of *PDE* transcripts can be regulated by the p54^nrb^/NONO nuclear protein, which is involved in transcription, splicing, and RNA export [20]. However, the mechanisms regulating the transcription and translation of different PDE3A isoforms, in addition to the function of each variant, are poorly understood.

In the present study, we characterized the pre-mRNA alternative promoter and splicing patterns of *PDE3A* variants to gain insights into the mechanisms regulating the expression of different isoforms of this gene. Specifically, we examined the mRNA levels of different isoforms in various human tissues and cells, and characterized the SFPQ-mediated transcriptional regulation of *PDE3A* gene expression. We found that *hPDE3A* expression was reduced in cervical cancer and the expression of *hPDE3A* increases the DNMDP sensitivity of cervical cancer cells.

## Experimental procedures

### Cell culture and transfection

The human cervical carcinoma HeLa cell line was maintained in Dulbecco’s modified Eagle’s medium (DMEM) supplemented with 10% heat-inactivated FBS (Gibco, NY, U.S.A.) at 37°C in a humid incubator with 5% CO_2_. Cells were transfected with plasmids and siRNAs using Lipofectamine 2000 (ThermoFisher Scientific, MA, U.S.A.) according to the manufacturer’s instructions.

### Plasmids, siRNAs, and reagents

The expression constructs for the PDE3A variants TSS1, TSS2, and TSS3 were obtained from cDNA of HeLa cells by PCR amplification. They were introduced into a pcDNA3 vector. The following forward PCR primers were used: *TSS1*, 5′-GCG CGA ATT CAT GGG CTT GTA CCT CCT GCG-3′; *TSS2*, 5′-GCG CGA ATT CAT GAT CGC CTT GAC TAG CGC-3′; *TSS3*, 5′-GCG CGA ATT CAT GTC CGG CTG CAG CAG CAA-3′. The reverse PCR primer used for all variants was: 5′-CCT TGC GGC CGC TCA CTG GTC TGG CTT TTG GG-3′. Underlined nucleotides represent adapter sequences, including the EcoRI/NotI restriction sites. PCR products were sequenced to verify expected gene amplification. A mixture of siRNAs against human *PDE3A* (sc-41592), human *SFPQ* (sc-38304), and human *NonO* (sc-38163) as well as control *siRNA-A* (sc-37007) were purchased from Santa Cruz Biotechnology (TX, U.S.A.). Actinomycin D was purchased from Sigma–Aldrich (MO, U.S.A.). DNMDP was purchased from the Pharmakon collection (MicroSource Discovery Systems, Inc., WA, U.S.A.).

### RNA preparation, RT-PCR, and quantitative PCR

Total RNA was isolated from cultured cells using the RNeasy Mini Kit (Qiagen, Hilden, Germany) and reverse transcribed with the SuperScript III Reverse Transcriptase (Thermo Fisher Scientific, MA, U.S.A.) using random hexamers. Reverse-transcription quantitative PCR (RT-qPCR) was performed using FastStart Universal SYBR Green Master Premix (Roche, CA, U.S.A.) and NEXpro™ qRT-PCR Master Premix (Genes Laboratories, Korea). The following primers were used: *PDE3A*, 5′-GAC AGC GAT GAG TCA GGA GA-3′ and 5′-TCT GAA GAG TGC GAC TGA GG-3′; *GAPDH*, 5′-CCA TGG AGA AGG CTG GGG-3′ and 5′-CAA AGT TGT CAT GGA TGA CC-3′. RT-qPCR was performed using the Applied Biosystems 7900HT Detection System. The specificity of the products generated with each set of primers was examined using agarose gel electrophoresis and confirmed by a melting curve analysis.

### Preparation of protein extracts and immunoblot analysis

Whole cell extracts were prepared using M-PER protein extraction reagent (Thermo Fisher Scientific) supplemented with a protease inhibitor cocktail (Roche, CA, U.S.A.). Extracted proteins were denatured and reduced by treatments with SDS and 2-mercaptoethanol, respectively. Proteins were then separated on 4–20% SDS-polyacrylamide gels (Bio–Rad, CA, U.S.A.) and transferred on to nitrocellulose membranes (Thermo Fisher Scientific, MA, U.S.A.). Immunoblot analysis was performed as described recently [21]. Anti-NONO, anti-SFPQ, and anti-ACTIN antibodies were purchased from Santa Cruz Biotechnology (TX, U.S.A.). Anti-PDE3A antibody was used as described previously [22].

### 5′-RACE

Human total RNAs were purchased from Origene (MD, U.S.A.); normal heart (R1234122-50, R1234122-P, and R1244122-50), cardiomyopathy (CR561340 and CR562037), normal cervix (CR559475, CR561069, and CR563004), and cervical adenocarcinoma (CR561176, CR561219, and CR559855). The Dynabeads mRNA Purification Kit (Thermo Fisher Scientific, MA, U.S.A.) was used to isolate mRNA, and 5′-RACE was performed using the 5′-RACE System for Rapid Amplification of cDNA Ends Kit (Thermo Fisher Scientific, MA, U.S.A.) according to the manufacturer’s instructions. First-strand cDNA was synthesized from polyadenylated mRNA using the following *hPDE3A* gene-specific primer: 5′-CAG AGC TCT CTT CAG AGT CAG AAC AGG-3′. Nested PCR was performed using the following *hPDE3A* gene-specific primer: 5′-CGT GGG CCT CGC CCA TGA CGG CGA TGT C-3′. PCR products were analyzed on agarose gels, and cloned into TA cloning vectors (Thermo Fisher Scientific, MA, U.S.A.) for sequencing. From this, the transcription start sites were determined.

### ChIP-PCR and electrophoretic mobility shift assay

ChIP was performed using reagents from Upstate Biotechnology Inc. (Lake Placid, NY) according to the manufacturer’s protocol. Immunoprecipitated DNA and input DNA were subjected to ChIP-PCR using primer sets that amplified the P1 and P2 regions of the *PDE3A* promoter, including the SFPQ-binding sites. Primer sequences were as follows: P1 forward, 5′-TTA TTT CTC TGA AAG AAG CAA GG-3′; P1 reverse, 5′-CTG GGT GGT TGT ACC ACC TTC TAG-3′; P2 forward, 5′-GGC ACC AGA CAT GTG GAA AC-3′; P2 reverse: 5′-GA ATC TTT GTA ATT TAC CTG TTG-3′. The SFPQ protein was synthesized *in vitro* from pCS3 + MT construct using the TNT Coupled Reticulocyte Lysate System for electrophoretic mobility shift assay (EMSA) [16]. ChIP-PCR product of the P1 region was end-labeled using [γ^32^P]ATP and T4 polynucleotide kinase. EMSA was performed as described previously [23].

### Cell viability after DNMDP treatment

HeLa cells were seeded at 5000 cells/well in 96-well plates. After incubating overnight, HeLa cells were transfected with PDE3A expression constructs for the TSS1, TSS2, and TSS3 variants. Twenty-four hours after transfection, cells were treated with 1 μM DNMDP for 20 or 30 h. After DNMDP treatment, cell viability was analyzed using CellTiter 96® AQueous One Solution Cell Proliferation Assay (Promega, WI, U.S.A.) based on MTS according to the manufacturer’s instructions. The absorbance was measured at 490 nm using the EMax Plus Microplate Reader (Molecular Devices, CA, U.S.A.). Cell viability was calculated by comparing values with those of controls.

## Results

### *PDE3A* mRNA expression decreased in SFPQ-depleted cells

**Figure 1:**
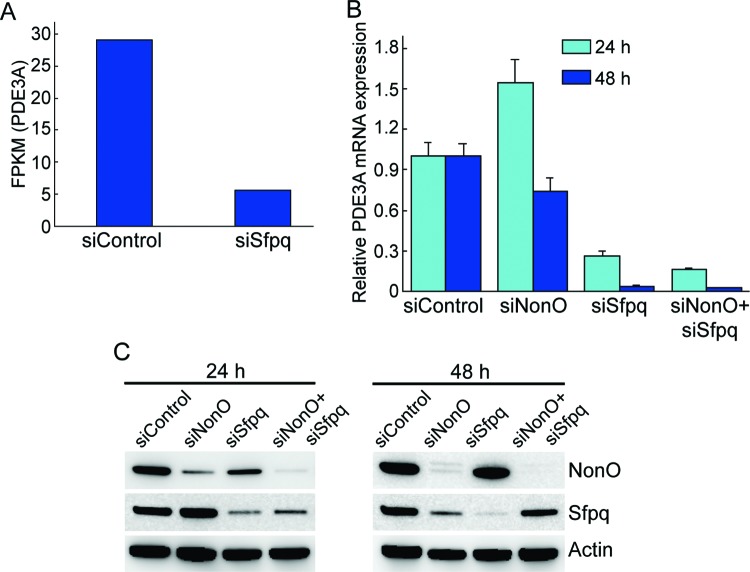
PDE3A expression correlates with SFPQ expression (**A**) *PDE3A* expression measured by RNA-seq in fragments per kb of transcript per million mapped reads (FPKM) in HeLa cells transiently expressing a SFPQ-targetting siRNA. (**B**) HeLa cells were transfected with the indicated siRNA. Total RNA was extracted at two different time points after siRNA transfection. The mRNA levels of *PDE3A* were measured by RT-qPCR and normalized to that of *GAPDH*. All results are expressed as the means ± S.E.M. for three independent experiments. (**C**) Analysis of protein expression by immunoblotting 24 and 48 h after indicated siRNA transfection.

While investigating the molecular targets of SFPQ using RNA-seq, *PDE3A* expression was found to be significantly decreased following SFPQ depletion (Figure 1A). To confirm this RNA-seq observation, we measured mRNA expression by RT-qPCR after SFPQ depletion or NonO depletion. In agreement with the RNA-seq results, SFPQ depletion decreased *PDE3A* expression after 24 and 48 h (Figure 1B). No significant change in the mRNA levels of *PDE3A* was observed after NonO depletion at 24 h, while the mRNA levels of *PDE3A* were slightly decreased after NonO depletion at 48 h. Immunoblot analysis confirmed that siRNA-mediated depletion efficiently reduced PDE3A expression (Figure 1C). These results suggest a role for SFPQ in the regulation of the *PDE3A* gene.

SFPQ contains regions rich in arginine/glycine and proline/glutamine close to the N-terminus [16]. It also possesses two RNA recognition motifs located in the C-terminus. SFPQ is a multifunctional nuclear protein that has been implicated in various aspects of the regulation of gene expression, including RNA splicing and transcription [24]. Thus, we investigated the molecular mechanisms of regulation of SFPQ-mediated *PDE3A* gene expression.

**Figure 2:**
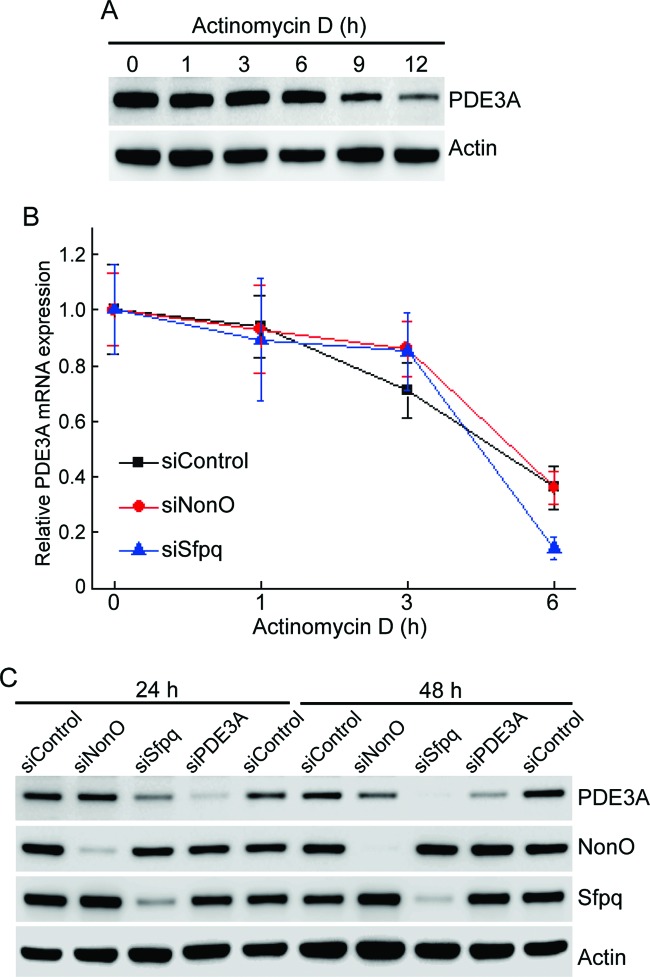
SFPQ transcriptionally regulates *PDE3A* (**A**) Analysis of protein expression by immunoblotting for PDE3A after actinomycin D treatment in HeLa cells for indicated time periods. (**B**) HeLa cells transfected with indicated siRNA were treated with actinomycin D (10 μg/ml). RNA was isolated at various time points after treatment, and mRNA expression levels for *PDE3A* were analyzed by RT-qPCR. All results are expressed as the means ± S.E.M. for three independent experiments. (**C**) Analysis of protein expression by immunoblotting 24 and 48 h after indicated siRNA was transfected into HeLa cells.

After treatment with the transcriptional inhibitor actinomycin D, a reduction in PDE3A protein levels was observed after 9 h (Figure 2A). Therefore, we measured *PDE3A* mRNA decay rate after depletion of SFPQ or NonO. There was no significant change in *PDE3A* mRNA decay rate following SFPQ or NonO depletion, suggesting that SFPQ transcriptionally regulates *PDE3A* (Figure 2B). To evaluate whether the decrease in PDE3A protein levels was accompanied by a relative change in the amount of SFPQ, PDE3A expression was measured by immunoblot analysis after either SFPQ or NonO depletion. A significant decrease in PDE3A expression was detected in SFPQ-depleted cells, but not in NonO-depleted cells (Figure 2C). These results suggest that SFPQ has a role in transcriptional regulation of *PDE3A*.

### Identification of the 5′-flanking region of human PDE3A

**Figure 3:**
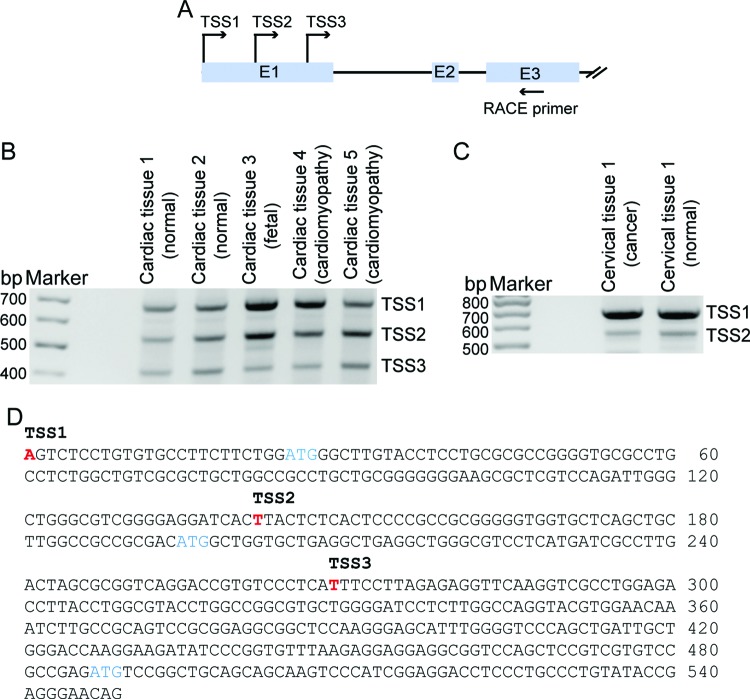
Identification of TSS for the human *PDE3A* gene (**A**) Schematic showing the first exon and flanking sequence for the human *PDE3A* gene. The TSSs for *PDE3A* were determined by 5′-RACE. (**B,C**) 5΄-RACE was carried out using RACE primers. Agarose gel analyses of the PCR products obtained from mRNA from the indicated tissues. (**D**) Sequences of a proximal 5′-flanking region of the *PDE3A* gene showing the TSSs.

To investigate the molecular mechanisms of SFPQ-mediated *PDE3A* transcription, 5′-RACE was carried out to identify a transcription start site (TSS) for human *PDE3A* using poly-A RNAs prepared from the heart and cervix. In heart, we found that *PDE3A* contains three TSSs within the first exon, each of which has a different level of gene expression (Figure 3A,B). Fetal and cardiomyopathy tissues showed stronger expression of *TSS1* and *TSS2* compared with adult normal cardiac tissues. Additionally, cervix expressed two TSSs, and displayed higher expression of *TSS1* in both normal and cancer tissues (Figure 3C). We speculated that each TSS uses different translation start codons (Figure 3D).

### Binding of SFPQ to the PDE3A promoter

**Figure 4:**
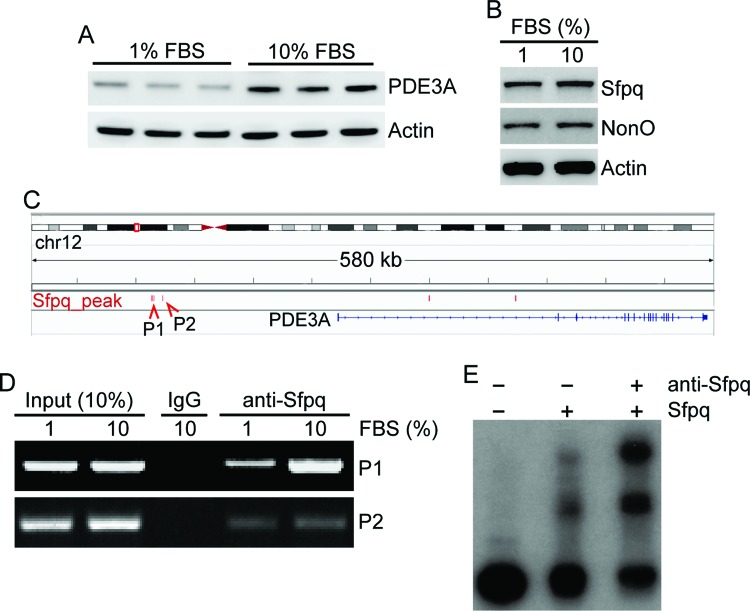
SFPQ binds the *PDE3A* promoter (**A,B**) Analysis of protein expression by immunoblotting for PDE3A, SFPQ, and NonO after HeLa cells were maintained in 1% FBS-enriched medium for 24 h. (**C**) ChIP sequencing (ChIP-seq) analysis of SFPQ binding sites in HeLa cells. Distribution of SFPQ binding sites (SFPQ peak, P1 and P2) within the *PDE3A* promoter on chromosome 12. (**D**) ChIP assay was carried using anti-SFPQ antibody on HeLa cells incubated in either 1 or 10% FBS-enriched medium for 24 h. PCR was performed using primers amplifying the P1 or P2 regions. Control IgG was used in parallel. PCR products were separated by agarose gel electrophoresis. (**E**) P1 DNA fragment of the PDE3A gene promoter which contains a putative SFPQ binding peak was analyzed using EMSA. Supershift assay was also carried out in which SFPQ antibody (anti-Sfpq) was added to the binding reaction.

For finding the expression level changes of PDE3A and the regulatory mechanism of SFPQ, we examined the changes of PDE3A expression under different concentrations of FBS. Since FBS provides growth-promoting factors in cell culture and affects the protein expression levels, the changes in PDE3A protein expression were examined by immunoblot analysis after three passages in 1 and 10% FBS treatment. FBS treatment (1%) decreased PDE3A protein levels, but did not affect SFPQ or NonO expression (Figure 4A,B). This suggests that PDE3A expression is regulated by more than the levels of SFPQ protein.

We hypothesized that SFPQ binding to the *PDE3A* promoter would be critical for its regulation. Thus, we investigated putative SFPQ binding sites in the *PDE3A* promoter using ChIP-seq. Three peaks of SFPQ binding within the *PDE3A* promoter were found at chr12:20673425-20673995 and chr12:20599778-20600539 for P1 and chr12:20373039-20373547 for P2 (Figure 4C). To confirm this, ChIP-PCR was carried out. We were unable to detect SFPQ binding at chr12:20673425-20673995 (results not shown). However, SFPQ strongly bound to the P1 region, and 1% FBS treatment reduced this binding (Figure 4D). The binding affinity of SFPQ to the P2 region was weaker than that to the P1 region in both samples. Further to the less binding of SFPQ to the P2 region was observed in 1% FBS-treated sample than 10% FBS-treated sample. In addition, we explored a physical interaction of SFPQ with P1 region using EMSA. Two slowly migrating complexes were formed with the SFPQ protein. Supershifted band was observed with an addition of anti-SFPQ antibody, suggesting that protein bound to P1 region is indeed SFPQ (Figure 4E). These results suggested that SFPQ directly binds to the *PDE3A* promoter and is regulated by serum concentration in the medium. Furthermore, SFPQ is critical for *PDE3* gene expression.

### PDE3A conferred sensitivity to DNMDP

**Figure 5:**
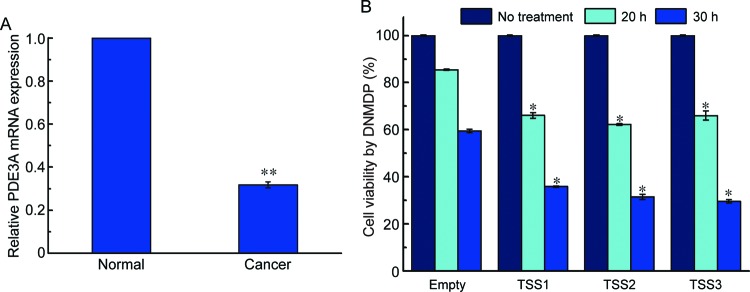
PDE3A promotes DNMDP-mediated cell death (**A**) *PDE3A* was measured by RT-qPCR and normalized to that of *GAPDH. PDE3A* mRNA levels were significantly decreased in cervical cancer tissues (Cancer) compared with normal cervical tissues (Normal). Data are presented as the mean values ± S.D. (*n*=3); **P<0.001 (Student’s *t* test). (**B**) HeLa cells were transfected with the indicated expression constructs of PDE3A variants. At 24 h post-transfection, cells were cultured with 1 μM DNMDP for the indicated time periods. Cell viability was measured by MTS assay and expressed in percentages. Data are presented as the mean values ± S.D. (*n*=3); *P<0.05 (Student’s *t* test).

A recent report showed that PDE3A affects the sensitivity of cancer cell lines to the anticancer therapeutic agent DNMDP [8]. To investigate whether *PDE3A* gene expression is altered in cervical cancer tissues, the expression levels of *PDE3A* mRNA were examined using RT-qPCR. The results showed that the relative expression of *PDE3A* mRNA in cervical cancer tissues was significantly lower compared with normal cervical tissues (Figure 5A). Next, we examined the effects of PDE3A on DNMDP sensitivity in HeLa cells by measuring cell viability after overexpression of PDE3A. The expression of each and all three PDE3A variants sensitized DNMDP-treated cells, suggesting that the expression of PDE3A indeed regulates DNMDP sensitivity in cervical cancer cells (Figure 5B).

## Discussion

In the present study, we identified SFPQ as a transcriptional regulator of the *PDE3A* gene. We demonstrated that SFPQ binding to the *PDE3A* promoter is essential for *PDE3A* expression and the binding of SFPQ is regulated by serum concentration. Serum contains a number of macromolecular proteins, low molecular weight nutrients, and other compounds necessary for cell growth such as hormones and attachment factors. The factors involved in the regulation of SFPQ binding to the *PDE3A* promoter remains to be elucidated. We also found that PDE3A expression is decreased in cervical cancer tissues compared with healthy tissues. In addition, we uncovered a role for PDE3A in DNMDP-mediated cell death.

The use of multiple promoters and TSSs is a common means of gene regulation; however, considerable variation and complexity in the patterns of alternative TSS usage exist. Alternative promoter usage can influence gene expression in numerous ways [25]. The overall amount of transcriptional initiation can vary amongst alternative promoters. The turnover or translation efficiency of mRNA variants with different leader exons can vary. Alternative promoters can have cell-type specificity, and can lead to the generation of protein isoforms differing at the N-terminus.

Based on our results, the three TSSs for *PDE3A* could theoretically generate three different N-termini. The first exon of *PDE3A* encodes a transmembrane domain that is critical for the protein function [26]. Thus, diverse N-termini in the transmembrane domain could be produced by alternative TSS usage. There is no obvious difference in the expression of the three PDE3A variants or in how they modulate DNMDP sensitivity in cervical cancer cells (Figure 5B). This suggests that the transmembrane domain of PDE3A is not responsible for regulating DNMDP sensitivity. We observed a similar pattern in PDE3A TSS expression between cervical tissues and HeLa cells (results not shown). Although two TSSs for PDE3A were identified in HeLa cells, immunoblotting with an anti-PDE3A antibody showed mainly one band at approximately 100 kDa. However, determining the mRNA variant of *PDE3A* and the TSS that produced the 100 kDa protein requires further study.

SFPQ is a ubiquitous and multifunctional nuclear protein essential for life in vertebrates [27,28]. SFPQ is a splicing factor associated with the polypyrimidine tract-binding protein [29]. It is implicated in various nuclear functions including splicing, RNA transport, DNA repair, and transcriptional regulation [24]. SFPQ binds DNA and RNA. It binds to promoters of a number of genes to regulate transcription [30–32]. Interestingly, the SFPQ protein expression level is often reduced in many types of cancer tissues when compared with healthy tissues (http://www.proteinatlas.org). In addition, SFPQ interacts with long non-coding RNAs in several cancer tissues [30,33–35]. However, there are no studies of the molecular function of SFPQ in cancer tissues. In this report, we found that serum concentration regulated SFPQ binding to the *PDE3A* promoter and resulted in decreased *PDE3A* levels without any change in SFPQ expression. Therefore, we speculate that allosteric modulation of SFPQ amongst other factors may affect its DNA-binding capacity in cancer cells, and changes in SFPQ expression are a critical means of regulating PDE3A expression.

The present study has uncovered a mechanism for PDE3A regulation. Recent reports suggested the potential role of PDE3A in modulating the effectiveness of the therapeutic agent DNMDP in cancer cells. Interestingly, we found that the expression level of *PDE3A* is lower in cervical cancer tissues compared with healthy control tissues (Figure 5A). In addition, our observation that PDE3A overexpression induced sensitivity to DNMDP in cervical cancer cells is similar to a recent report [8]. Further study is needed to understand how PDE3A influences DNMDP sensitivity.

## Abbreviations

EMSA: electrophoretic mobility shift assay
HDL: high-density lipoprotein
NONO: non-POU domain-containing octamer-binding protein
PDE: phosphodiesterase
PDE3A: phosphodiesterase 3A
RACE: 5′-rapid amplification of cDNA end
RT-qPCR: reverse-transcription quantitative PCR
SFPQ: splicing factor proline and glutamine rich
TNF: tumor necrosis factor
